# Evaluation of the implementation effect of pre-audit of inpatient medical orders: based on the ORTCC model

**DOI:** 10.3389/fphar.2025.1681245

**Published:** 2025-10-01

**Authors:** Xiaoli Qin, Shanhong Luo, Heng Xi, Min Xu, Yujie Yang, Qin He

**Affiliations:** ^1^ Department of Pharmacy, Affiliated Hospital of Southwest Jiaotong University, The Third People’s Hospital of Chengdu, Chengdu, Sichuan, China; ^2^ Department of Pharmacy, Affiliated Hospital of Sichuan University, Chengdu Second People’s Hospital, Chengdu, Sichuan, China

**Keywords:** adverse drug events, medication errors, prescription automatic screening system, ORTCC model, clinical decision support system

## Abstract

**Objective:**

This study aimed to evaluate the impact of implementing a pre-audit system for inpatient medical orders based on the ORTCC (Objectives, Rules, Training, Check, Culture) management model in a tertiary hospital in Chengdu, China. The primary goals were to enhance the pass rate of medical orders, reduce medication errors (MEs), and improve patient safety regarding medication administration.

**Methods:**

A pre-post intervention study was conducted using data from 2022 (pre-implementation) and 2024 (post-implementation). The Prescription Automatic Screening System (PASS) was employed to analyze medical orders, incorporating a “three review and three interception” model involving system alerts, pharmacist reviews, and dispensing checks. Key metrics included the qualification rate of medical orders, physician modification rates, and types of unreasonable orders. Statistical analysis was performed using SPSS (version 27), with chi-square tests for categorical data.

**Results:**

Following implementation, unreasonable medical orders significantly decreased from 540,000 in 2022 to 79,514 in 2024. The physician modification rate increased from 8.59% to 31.86% (*P* < 0.001), while the final qualification rate improved by 21.31% (*P* < 0.001). Modules with frequent issues (e.g., dosage, administration routes, drug compatibility) showed reduced error proportions (*P* < 0.05). Targeted interventions in high-risk departments (e.g., cardiovascular, ICU) further reduced errors (*P* < 0.05).

**Conclusion:**

The ORTCC-based pre-audit system significantly enhanced the rationality of medical orders, reduced MEs, and promoted safer medication practices. Continuous pharmacist training, dynamic rule updates, and advanced technologies are recommended to sustain improvements and address system limitations, such as false alerts.

## 1 Introduction

Medication errors (MEs) are any preventable event that can cause inappropriate medication use or harm to a patient, according to the National Coordinating Council for Medication Error Reporting and Prevention (NCC MERP). This definition applies while the medication is in the possession of a healthcare professional, patient, or consumer (Edwards and Aronson, 2000; Classen et al., 2005; Ciapponi et al., 2021). In the United States, 53%–58% of adverse drug event (ADE) related hospitalizations were due to MEs. MEs are estimated to account for approximately 7,000 deaths annually; they represent 1 in 854 inpatient deaths and 1 in 131 outpatient deaths (Elshayib and Pawola, 2020). Meanwhile, the deaths of more than 2.5 million patients in approximately 50 million hospitalized patients each year in China are related to drug-induced adverse reactions (Ji et al., 2019). Research showed that approximately half of all MEs occurred at the stage of drug ordering (Kaushal et al., 2003). The main types of MEs include missed doses and incorrect dosages, frequencies, or routes of administration. The frequency and type of MEs identified often vary based on the detection methods. Research indicated that computerized physician order entry (CPOE) systems and clinical decision support systems (CDSS) can reduce MEs (Prgomet et al., 2017; Roumeliotis et al., 2019). CPOE systems vary; some provide medication lists, while others check for drug interactions, allergies, and lab-based prescription assessments (Ammenwerth et al., 2008).

Pharmacists play a vital role in preventing drug-related problems (DRPs) in both ambulatory care and hospital settings (Kaboli et al., 2006; Knudsen et al., 2007; Guignard et al., 2015). They are responsible for advising healthcare professionals, educating patients, and reviewing medications to ensure the quality of medicines provided to patients (Schnipper et al., 2006; Blouin and Adams, 2017; Bouchaud et al., 2022; Zaij et al., 2023). As national policies evolve, the daily responsibilities of pharmacists are changing, moving away from a product-focused approach to a patient-centered model of care (Kopp et al., 2007). In June 2018, China issued new prescription review specifications for medical institutions, outlining the principles of prescription review and emphasizing the pharmacist’s primary role in this process. Pharmacists must review all prescriptions and medical orders before processing payment and dispensing medications (Ji et al., 2019).

The pre-prescription review has emerged as a new model for hospitals in recent years, promoting patient safety and the rational use of medications compared to traditional prescription reviews. During the initial phase of the work, an automatic prescription Screening System (PASS) was officially launched in 2019, and the pre-approval process for prescriptions was officially launched in January 2020. The results indicated a marked improvement: the pass rate for outpatient prescriptions increased from 84% in 2019 to 97% in 2020, demonstrating significant success. Building on this foundation, in 2023, our hospital implemented a pre-audit of inpatient medical orders based on the ORTCC (Objectives, Rules, Training, Check, and Culture) management model. This study evaluated key indicators, such as the pass rate of medical orders and the incidence of unreasonable issues, both before and after implementing the pre-audit system. This aims to enhance the efficiency of prescription reviews further, standardize the rational use of drugs, reduce MEs caused while prescribing medical orders, and identify areas for improvement and actionable measures.

## 2 Materials and methods

### 2.1 Medical records

This study was conducted at a grade III tertiary hospital in Chengdu, China. We initiated a pre-audit of inpatient medical orders in 2023, implementing this process departmentally and in batches. By the end of 2023, we aim to achieve full coverage of hospitalization audits. In this study, we utilized the Hospital Information Management System (HIS) and PASS to query and analyze data on the qualification rate of inpatient orders before and after the pre-audit process. The data analyzed was from 2022 (before the system launch) and 2024 (after the system launch). We also examined the modification rate of doctors’ orders following the system audit and the proportion of unreasonable order types.

### 2.2 Methods

The ORTCC model serves as the theoretical foundation for refined management, comprising five key elements: Objectives, Rules, Training, Check, and Culture.

#### 2.2.1 Set objectives

To enhance the rationality of medical orders, improve the pass rate of these orders, reduce MEs caused while prescribing medical orders, and ensure patient medication safety.

#### 2.2.2 The establishment and deployment of the medical order review system rules

**FIGURE 1:**
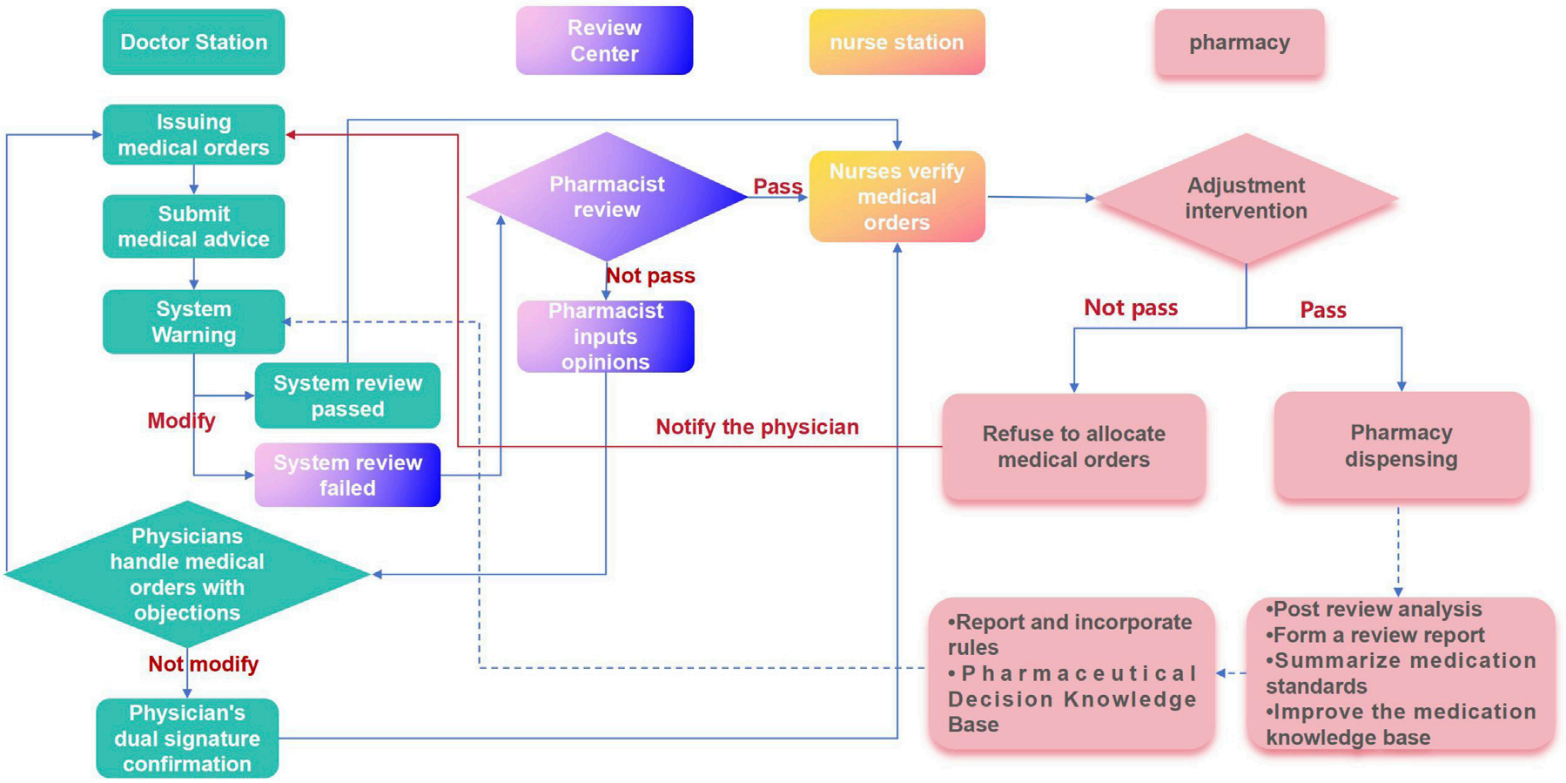
Inpatient medical order review process.

A medical order review working group comprising clinical pharmacists, full-time prescription review pharmacists, dispensing pharmacists, and information pharmacists should be established. This group will establish and maintain a process for reviewing inpatient medical orders and associated rules. We employ a “three review and three interception” model for medical order review (Figure 1). Initially, the PASS system intercepts physician orders for an initial review. If the system identifies an order as potentially unreasonable, it is returned to the physician for modification or review. Subsequently, a reviewing pharmacist conducts a second review to differentiate between true and false positive alerts. Only pharmacist-approved medical orders are then transferred to the nurse station. Finally, when all medical orders, including those initially misdiagnosed or falsely negative, are processed for inpatient dispensing, the dispensing pharmacist conducts a third and final review to intercept potential errors.

**FIGURE 2:**
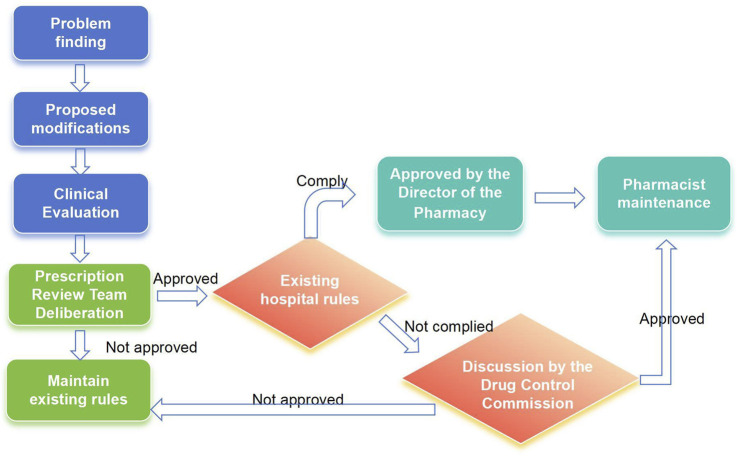
Rule maintenance process.

Pharmacists can apply to modify audit rules found to be missing in the system during the review, evaluation, and pharmaceutical monitoring of medical orders, as well as audit rules that clinical departments consider unsuitable for daily medical work. The applicant for a rule modification should consult relevant laws, regulations, and evidence-based medicine resources. They must then conduct an evidence-based evaluation of the proposed rule modification. The medical order review working group will then review these evaluation opinions. If approved, the rule maintenance pharmacist will modify the rules. If the review fails, the existing rules will be maintained (Figure 2). This medical practice evidence evaluation of the modification basis should include an assessment of effectiveness, recommendations, and evidence levels, all grounded in evidence-based medicine principles. The PASS utilizes a dynamic, evidence-based knowledge base, incorporating product information, clinical guidelines and conventions, clinical pathways, and national formularies. The evaluation results of this medical practice evidence determine our hospital’s refined maintenance principle.

The PASS system categorizes warning issues into black, red, orange, and yellow alerts based on risk level, with black alerts indicating the highest risk. We have directly blocked the red and black lights in the drug administration routes and frequencies, bypassing the pharmacist’s review. Medical orders that fail system review must be returned to the physician for modification. Meanwhile, Warnings below the red alert level in the drug contraindication module are intended for physician review, while black alerts require consultation with a pharmacist for professional judgment. Table 1 details the processing path for inpatient medical orders.

**TABLE 1:** System warning problem handling path.

Module	Black light warning issue			Red light warning issue			
	Intercept	Manual review	Exempt	Intercept	Manual review	Exempt	Only reminding
Super indication			◎			◎	
Total parenteral nutrition			◎			◎	
Drug allergy	☒			☒			
Drug contraindications		★					▲
Drug interactions		★					▲
Route of administration	☒			☒			
Medication frequency	☒			☒			
Dose range		★			★		
Repeated medication		★			★		
Adverse reactions		★			★		
Medication for special populations		★			★		
A dose of liver and kidney damage		★			★		
Compatibility concentration		★			★		
*In vitro* compatibility		★			★		

☒ The improper handling method for such medical orders is that the system automatically intercepts them and does not require review by pharmacists. Only the physicians can make corrections.

★ The inappropriate prescription handling method stipulated in this article is that the physician can request the pharmacist to review it. The pharmacist will judge based on professional knowledge as to whether it is reasonable.

◎ The inappropriate medical advice handling method stipulated in this rule can be retained by default, but clinical pharmacists or dispensing pharmacists will review it in the future.

▲ The inappropriate handling method for such medical orders merely alerts the physician. Based on the patient’s condition, the physician can decide whether to continue using or monitor it.

#### 2.2.3 Building a training system

Clinical pharmacists and department liaison pharmacists offer personalized training to department staff, ensuring they become proficient in system operation. This training also facilitates effective communication and the exchange of feedback or medical advice on relevant issues.

#### 2.2.4 Check the assessment system

Every month, we review approximately 3,000 hospitalization records, covering types 1 and 2 incisions, key monitoring of medications, etc. Based on established drug review standards, clinical pharmacists comprehensively evaluate the rationality of medication indications and treatment duration. This evaluation is particularly important for reviewing medical orders flagged by the system when pharmacists are offline. The pharmacists also organize and continuously update the system’s rules to correct imperfections. The medical department reviews these evaluation results quarterly, publishes them regularly, and discloses any identified unreasonable medical orders. These findings serve as a basis for penalties, which are incorporated into the performance evaluations and annual assessments of relevant departments and staff.

#### 2.2.5 Creating a cultural system

Integrating the concept of pre-control into rational drug use involves more than just evaluating prescription rationality. Effective management requires clarifying the responsibilities at each level of pharmaceutical oversight. Furthermore, it is crucial to provide in-depth clinical training for doctors on rational drug use. Before going online, we provided systematic training for all physicians and pharmacists in each department. Moreover, simultaneously, a robust, cyclical process of supervision, evaluation, feedback, intervention, and repeated cycles will enable doctors to master the PASS system’s prescription norms and promote rational drug use.

### 2.3 Statistical analysis

The SPSS software (IBM, version 27) was used for all statistical analyses. Descriptive statistics (number and frequency) were performed to assess system audit tasks, the rate of unreasonable system audits, the number of intercepted system tasks, and the doctor modification rate after system audits. Group comparisons were conducted using the chi-square test. Statistical significance was defined as a *P* < 0.05.

## 3 Results

### 3.1 Comparison of key indicators before and after the implementation of the plan

In 2022 and 2024, the total system audit tasks reached approximately 2.1 million. The system experienced a significant reduction in unreasonable tasks following the implementation of the plan. In 2022, the system identified 540,000 unreasonable tasks. Following the plan’s implementation, this number decreased to only 79,514 for the entire year, representing a reduction of approximately 460,000. Post-implementation, the modification rate of medical orders significantly increased, from 8.59% in 2022 to 31.86% in 2024, *P* < 0.001. The final qualification rate increased 21.31%, *P* < 0.001 (Table 2).

**TABLE 2:** Comparison of key indicators before and after the implementation of the plan.

Items	2022	2024	Difference	*P*
Total number of system audit tasks (n)	2,108,784	2,188,277	79,493	0.451
Number of unreasonable tasks reviewed by the system (n)	547,499	79,514	−467 985	<0.001
Unreasonable rate of system review (%)	25.96	3.67	−22.29	<0.001
Doctor modification rate after system review (%)	8.59	31.86	23.27	<0.001
Qualified rate of medical orders (%)	76.38	97.69	21.31	<0.001

### 3.2 Comparison of unreasonable problem types before and after the plan’s implementation

After reviewing medical orders, the modules with the most frequent issues are indications, administration routes, *in vitro* compatibility, and repeated use. As demonstrated in Table 3, the proportion of unreasonable issues in these modules decreased after implementing the comparative plan, *P* < 0.05.

**TABLE 3:** Comparison of unreasonable problem types before and after the plan’s implementation.

Question type	Review point	2022	2024	Difference	*P*
		Proportion of total problems (%)			
Dose range	Medication frequency	15.08	6.28	−8.8	0.022
	Each dose	11.56	8.54	−3.02	
	Daily dose	10.19	8.85	−1.34	
Route of administration	Route of administration	12.48	8.30	−4.18	
*In vitro* compatibility	*In vitro* compatibility	12.28	2.17	−10.11	
Repeated medication	Repeated medication	10.33	9.05	−1.28	

### 3.3 Classification of system audit intervention before and after plan implementation - top 10 departments

A review of medical orders identified the cardiovascular and cerebrovascular ward, ICU, and respiratory ICU as the top 10 departments with the highest number of unreasonable orders. Following this finding, the project will prioritize interventions in these specific wards. Table 4 demonstrates a decrease in unreasonable medical orders compared to 2022. Among these 10 departments, except for the slight difference in the total number of tasks (*P* > 0.05), the improvements in the number of unreasonable tasks, the number of tasks modified by doctors, the modification rate, and the qualified rate of medical orders were all significant (*P* < 0.05).

**TABLE 4:** Classification of system audit intervention before and after plan implementation - top 10 departments.

	Total tasks (n)			Unreasonable tasks (n)			Unreasonable rate (%)			Tasks modified(n)			Modification rate (%)			Task qualification rate (%)		
	Before	After	D	Before	After	D	Before	After	D	Before	After	D	Before	After	D	Before	After	D
Cardiovascular Surgery ICU	26,043	23,558	−2,485	15,878	960	−14,918	60.97	4.08	−56.89	3,230	170	−3,060	20.34	17.71	−2.63	52.2	97.25	45.05
ICU	158,187	107,679	−50,508	94,083	3,824	−90,259	59.48	3.55	−55.93	9,653	1,070	−8,583	10.26	27.98	17.72	46.38	97.65	51.27
Cardiovascular and Vascular Surgery	30,542	25,540	−5,002	15,650	960	−14,690	51.24	3.76	−47.48	682	432	−250	4.36	45	40.64	50.9	98.09	47.19
Department of Rehabilitation Medicine	23,565	15,033	−8,532	10,131	619	−9,512	42.99	4.12	−38.87	1,172	288	−884	11.57	46.53	34.96	62.69	97.9	35.21
Respiratory ICU	45,773	34,606	−11,167	17,875	1,180	−16,695	39.05	3.41	−35.64	2,600	223	−2,377	14.55	18.9	4.35	66.85	97.34	30.49
Nephrology Department	73,321	65,134	−8,187	24,904	2,989	−21,915	33.97	4.59	−29.38	2,197	1,063	−1,134	8.82	35.56	26.74	69.45	97.43	27.98
Gastrointestinal surgery	20,413	16,308	−4,105	6,888	404	−6,484	33.74	2.48	−31.26	177	135	−42	2.57	33.42	30.85	67.2	98.63	31.43
Neurosurgery	41,562	32,432	−9,130	13,010	1869	−11,141	31.3	5.76	−25.54	354	462	108	2.72	24.72	22	69.76	95.89	26.13
Oncology Department 1	48,592	46,728	−1,864	14,862	1,487	−13,375	30.21	3.18	−27.03	1,362	702	−660	9.28	47.21	37.93	74.21	98.47	24.26
Oncology Department 2	75,243	53,040	−22,203	21,230	1,258	−19,972	28.22	2.37	−25.85	2,646	602	−2,044	12.46	47.85	35.39	75.48	98.97	23.49
*P*	0.446			0.014			<0.001			0.045			<0.001			<0.001		

## 4 Discussion

In 2020, driven by China’s ongoing medical reforms and the demand for high-quality pharmaceutical services, we implemented the PASS system. It significantly improves over traditional prescription review methods due to its rapid response time, averaging approximately 5 s per task, substantially faster than manual review. Furthermore, the PASS system provides a comprehensive and precise audit scope, encompassing patient-specific factors such as age, gender, and diagnosis, as well as therapy regimens including medication selection, dosage, frequency, route of administration, and potential drug-drug interactions. Following the implementation of this pre-validation system, we observed a marked improvement in the pass rate of outpatient prescriptions, demonstrating its positive impact.

To enhance drug quality control and minimize MEs, this study implemented the pre-audit of inpatient medical orders based on the ORTCC model, across five key aspects: Objectives, Rules, Training, Check, and Culture. The effectiveness of this pre-audit system for medical orders was evaluated by comparing data before and after its implementation. The results demonstrate a significant decrease in unqualified medical order tasks in hospitalization, from 547,499 in 2022 to 79,514 in 2024. Concurrently, the qualified rate of inpatient medical orders significantly improved, rising from 76.38% in 2022 to 97.69% in 2024 (*P* < 0.001), indicating statistical significance. Moreover, the system effectively addressed frequently occurring issues in medical orders. Modules with a high prevalence of problems, such as dosage range, administration route, *in vitro* compatibility, and repeated use, showed a reduced proportion of unreasonable issues after the system’s implementation. Notably, the administration route and frequency presented a unique case. Communication with physicians revealed that these errors were primarily due to oversight. Consequently, the system was configured to directly intercept these specific errors without requiring pharmacist review, streamlining the workflow for pharmacists and preventing potentially unreasonable orders from being missed during offline periods.

Furthermore, analysis of the top 10 departments with the highest number of unreasonable medical orders before system implementation showed a significant reduction in unreasonable audits and task irrationality rates (*P* < 0.05), demonstrating statistical significance. While the number of audit tasks remained relatively stable, the doctors’ modification rate doubled, suggesting a heightened awareness and correction of errors. Overall, the plan led to a noticeable change in doctor behaviour; previously, system prompts were often ignored. However, post-implementation, the modification rate of medical orders significantly increased, from 8.59% in 2022 to 31.86% in 2024, *P* < 0.001. These findings suggest that the PASS system has significantly enhanced the rationality of medical orders, positively impacting rational drug use in the hospital’s clinical practice. This ultimately ensures safer medication practices for patients and reduces the incidence of MEs.

The PASS system requires comprehensive drug information, including usage and dosage, adverse reactions, incompatibilities, and interactions, supported by big data. This software is updated annually with data from guidelines and expert consensus. However, a qualified prescription review software should allow users to personalize the configuration, incorporating information from sources like instructions, clinical trials, and post-clinical demonstration data on rational medication use. Over time, rational drug software will continuously improve and integrate further into hospitals (Liu et al., 2021). By the end of 2024, we have manually maintained approximately 90,000 rules to personalize the system and better align it with clinical needs. A key aspect of inpatient diagnosis logic is that it reflects the patient’s condition upon admission, not necessarily their current state. For instance, a patient admitted with gastrointestinal bleeding may require discontinuing nonsteroidal anti-inflammatory drugs. However, the system retains the gastrointestinal bleeding diagnosis even after the bleeding resolves or the patient is discharged. To address this, we have configured the drug contraindication module with different warning levels. A red light warning serves as a prompt for the doctor, while a black light warning triggers a review by the pharmacist. The pharmacist then conducts a detailed review of the medical record to determine the appropriateness of the drug. Similarly, for medications prescribed without clear indications, we bypass pre-hospitalization review and instead rely on continuous drug rationality review by clinical pharmacists. Moreover, a dedicated pharmacist in the intravenous configuration center reviews parenteral nutrition orders, which the PASS system does not intercept separately. These personalized rule settings enhance patient medication safety, reduce pharmacist workload, minimize unnecessary alerts for doctors, and improve the system’s overall usability.

After applying the pre-audit of inpatient medical orders, the relative number of irrational medical orders was reduced. However, the errors brought about by the system itself cannot be ignored. It is crucial to consider the possibility of false negative alerts and false positive medical orders and the risk of alert fatigue when frequent, clinically irrelevant alerts are ignored. Therefore, frontline pharmacists should perform a final check (Hincapie et al., 2014).

Pre-audit review is a strategy to improve medical service quality by promoting rational drug use. For example, pharmacists must assess the reasonableness of the system’s daily dose limit reminders, especially when patients have long-term and temporary medical orders. Furthermore, if a child’s single dose is calculated based on their weight in kilograms, it may exceed the specified dosage of the drug. Consequently, pharmacists must carefully review and examine these calculations during the review process. By integrating the ORTCC management model with the PASS system, we conducted a pre-audit of inpatient orders, resulting in more closed-loop management of rational drug use. There are ORTCC models available for other medical quality research in the market, as well as studies related to pre-hospitalization review. However, integrating the ORTCC model with PASS is not available. This is also a novelty of this research.

Our study enhances pharmacists’ efficiency and accuracy, and proposes new ideas for improving the rational use of drugs among patients. Nevertheless, several limitations should be acknowledged. First, relying solely on single-center studies is insufficient; more data are needed for verification. In addition, PASS technology may introduce errors. The system still has issues with false negatives and false positives, such as incorrect dosing or route recommendations. To mitigate these risks, we plan to utilize more data, implement frequent updates, and adopt advanced technologies to enhance the accuracy of the review system.

## 5 Conclusion

Effective management of MEs and rational drug use are important global issues. The integration of the ORTCC model and the PASS system has improved inpatient prescription practices and ensured patient medication safety. To enhance drug use and patient safety, it is recommended that these systems be incorporated into the healthcare framework.

## Data Availability

The original contributions presented in the study are included in the article/supplementary material, further inquiries can be directed to the corresponding author.

## Abbreviations

ME, medication error; ADE, adverse drug event; NCC MERP, National Coordinating Council for Medication Error Reporting and Prevention; CPOE, computerized physician order entry system; CDSS, clinical decision support system; DRP, drug-related problem; PASS, prescription automatic screening system; HIS, hospital information management system.
